# Increased risk of deep neck infection among HIV-infected patients in the era of highly active antiretroviral therapy—a population-based follow-up study

**DOI:** 10.1186/1471-2334-13-183

**Published:** 2013-04-22

**Authors:** Ching-Feng Liu, Shih-Feng Weng, Yung-Song Lin, Chih-Sheng Lin, Ching-Feng Lien, Jhi-Joung Wang

**Affiliations:** 1 Department of Otolaryngology, Chi Mei Medical Center, Tainan, Taiwan; 2 Department of Biological Science and Technology, National Chiao Tung University, Hsinchu, Taiwan; 3 Department of Medical Research, Chi Mei Medical Center, Tainan, Taiwan; 4 Department of Hospital and Health Care Administration, Chia Nan University of Pharmacy and Science, Tainan, Taiwan

**Keywords:** Deep neck infection, HIV, Highly active antiretroviral therapy

## Abstract

**Background:**

Deep neck infections (DNIs) in HIV-infected patients often produce severe complications, even death. Data on the incidence rates and risks of DNI among HIV-infected patients are scarce, particularly with the widespread use of highly active antiretroviral therapy (HAART). We evaluated the incidence rates and risks for DNI among HIV-infected patients and observed the long-term trends.

**Methods:**

A total of 9888 new HIV-infected patients diagnosed in 2001–2007 were included and matched with 49440 randomly selected subjects. The HIV-infected subjects were offered free access to HAART. All subjects were traced until December 2009. A Kaplan-Meier analysis generated the cumulative DNI incidence rate. The adjusted hazard ratio was computed using Cox proportional hazard regressions.

**Results:**

From the HIV-infected and comparison cohorts, 222 individuals (57.01 cases per 10000 person-years) and 735 individuals (35.54 cases per 10000 person-years) developed DNI, respectively. The log rank test indicated that patients with HIV had a significantly higher 8-year incidence rate of DNI than the control group (*P* < 0.0001). The adjusted hazard ratio for developing DNI after an HIV attack during the mean 3.94 years follow-up period was 1.59. The incidence rate and relative risk of DNI were 74.58 (per 10000 person-years) and 2.05 (*P* < 0.0001). Both figures were highest in the first follow-up year and decreased year-by-year thereafter.

**Conclusion:**

The risk of developing DNI is significantly elevated among HIV-infected patients, even with free access to HAART. Additional research is needed to examine the role of HAART in reducing the risk.

## Background

Deep neck infection (DNI) is a serious disorder defined as infections that track along the facial planes of the head and neck, which may result in abscess formation or cellulitis as a result of bacterial invasion. This infection usually invades from other primary sites, usually within the pharynx or oral cavity. The submandibular (and sublingual), parapharyngeal, and retropharyngeal spaces are the most clinically relevant spaces in the neck. These spaces also connect to other important structures such as the mediastinum, carotid sheath, skull base and meninges in the head, and neck and thorax, providing pathogens with easy access. Once infection reaches these important areas, mortality rates can be as high as 20–50% [1].

Deep neck infection is one of the most lethal infections and can occur at any age and result in life-threatening complications. In previous studies, the predisposing risk factors for DNI included diabetes mellitus, drug abuse, congenital neck cysts, age, and oral hygiene. Francisco Vieira *et al.* suggested that immune suppression diseases, such as chronic renal disease and hepatic disease, and chronic steroid therapy for autoimmune disease could be risk factors for DNI [2-8]. In Srivanitchapoom’s study, they emphasized that clinical physicians were required to make certain assessments because of the potential for complications when DNI was diagnosed in patients with compromised immune systems [9]. The human immunodeficiency virus (HIV) epidemic is one of the most important public health problems of this century and has caused the deaths of a significant number of immune-compromised patients. The immune deficits of HIV-infected patients are broader and include both abnormalities in humoral immunity and , in the late stages of disease, neutrophil function .As a result, bacterial infections frequently complicate HIV disease [10] In an analysis of published reports on bacterial pneumonia, rates were up to 25-fold higher among HIV-infected adults than in the general population [11]. Consequently, it is reasonable to project that more and more HIV-infected patients will suffer from DNI as their immune systems gradually decline. One previous study indicated that immune-compromised patients are more apt to develop complicated and life-threatening DNI as compared to normal people [9]. However, the sample size was small and the risk of DNI among HIV-infected patients was still unknown, especially in the era of highly active antiretroviral therapy (HAART).

Although highly active antiretroviral therapy (HAART), the cornerstone of treating HIV infection, has been proven effective at decreasing mortality, morbidity, opportunistic infections, and hospitalization for HIV-infected patients [12-14], it is not known whether HAART will also work to decrease the risk of DNI. The Taiwanese government has provided all HIV-infected citizens with free access to HAART from April 1997 to the present day, making it possible to study the effect of widespread use of HAART on the risk of developing DNI among HIV-infected patients.

To our knowledge, despite some case reports, large sample data regarding the exact frequency and risk of DNI among HIV-infected patients are still lacking. Our study’s goal was to investigate the risk of DNI among HIV-infected patients when free access to HAART was available.

## Methods

### Database

This study used a data set released by the Taiwan National Health Research Institute in 2011. Taiwan’s National Health Insurance program was instituted in March 1995 and provides coverage for over 98% of the residents. The National Health Research Institutes transferred national health insurance reimbursement data into files for research. These files provided the detailed health care services information for each patient, including all payments for outpatient visits, hospitalizations, and prescriptions. For each outpatient visit or hospitalization, the data contained up to 3–5 diagnoses coded under the *International Classification of Diseases, Ninth Revision*, along with the prescription drugs and doses, special treatments (such as surgery), and dates of these orders. This study was conducted according to the Declaration of Helsinki and was approved by Institutional Review Boards (IRBs) at Chi Mei Medical Center (10109-E01).The IRBs waived the need for informed consents (written and oral) from the participants because the data set used in this study consists of nationwide, unidentifiable, secondary data released to the public for research purposes. This waiver does not adversely affect the rights and welfare of the subjects.

### Study sample

The study cohort was comprised of all the patients, from January 1, 2002 to December 31, 2007, who were newly diagnosed for treatment of HIV infection in more than 3 outpatient visits or in 1 hospitalization claim in Taiwan (*International Classification of Diseases, 9th Revision, Clinical Modification* Code 042–044, V08 ). Since DNI was defined as a severe infection in the deep neck space, patients assigned ICD-9-CM codes 528.3 (cellulitis and abscess of oral soft tissues; Ludwig angina), 478.22 (parapharyngeal abscess), 478.24 (retropharyngeal abscess), and 682.11 (cellulitis and abscess of neck), as well as patients with peritonsillar abscesses, sialoadenitis, salivary gland abscesses, superficial abscesses, and facial abscesses were excluded. Patients who had been diagnosed with DNI before their HIV infection or had a history of congenital neck cysts (such as thyroglossal duct and branchial cleft cysts) were also excluded. Finally, a total of 9888 HIV-infected patients were included in this study.

### Control group

For each HIV case, five controls without HIV were randomly selected in 2000 from the longitudinal Health Insurance Database 2000, a data subset of the National Health Insurance Research database (NHIRD) that contains all the claim data (from 1996 to 2009) for one million beneficiaries (4.34% of the total population). The controls were matched by gender, age, and index date. The index date for the HIV patients was the date of their first registry and the index date for the controls was created by matching the date of the HIV subject’s index date. As with the HIV patients, the patients diagnosed with DNI before the index date were excluded.

Each patient was followed up to determine the incidence of DNI until the end of 2009 or was censored because of death. Incidence rates (per 10000 person years), incidence rate ratios (IRRs), and hazard ratios (HRs) for DNI were analysed. Comorbidities were also defined based on the claims data, including drug abuse (ICD-9-CM codes 304–305 and 965.00–965.09), diabetes mellitus (ICD-9-CM codes 250.00–250.90), liver cirrhosis (ICD-9-CM codes 571.2, 571.5–571.6), chronic kidney disease (ICD-9-CM codes 585–586), and autoimmune disease (ICD-9-CM codes 279.4, 710, 711.1–711.2, and 714). We included these comorbid conditions if the condition occurred either in the inpatient setting or in two or more ambulatory care claims coded 12 months before the index medical care date.

### Statistical analysis

Descriptive statistical analyses using Pearson χ^2^ tests were done to compare the HIV group and the control group in terms of sociodemographic characteristics and comorbidities. The incidence rate was calculated as the number of DNI cases identified during the follow-up, divided by the total person-years for each group by sex, age, and follow-up months. The risk of getting DNI was compared between the HIV group and the non-HIV group by estimating the incidence rate ratio using Poisson regression. Adjusted HRs for getting DNI were also estimated by Cox proportional hazard models. Kaplan-Meier analysis was used to calculate the cumulative incidence rates of DNI in the two cohorts, and the log rank test was used to analyse the differences between the survival curves. Kaplan-Meier analysis was also used to estimate the survival rate for HIV-infected patients in the DNI and no-DNI groups. Moreover, Pearson χ^2^ tests were done to compare the pattern of losses follow up between study cohorts and control cohorts in the each year to show whether the follow up was similar or not. All analyses were performed using SAS software, version 9.3 (SAS Institute, Cary, NC), and the statistical significance level was set at two-sided *p* < 0.05.

## Results

Table 1 describes the distribution of demographic characteristics and select comorbidities for both the study and comparison cohorts. After matching for sex and age, the results demonstrate that patients with HIV were more likely to have the comorbidities diabetes mellitus (*P* = 0.0106), drug abuse (*P* < 0.0001), chronic renal failure (*P* = 0.0005), liver cirrhosis (*P* < 0.0001), and autoimmune disease (*P* < 0.0001) than the control groups.

**Table 1:** Demographic characteristics and comorbid medical disorders for HIV patients and comparison group patient in Taiwan

	**HIV (N = 9888)**	**Controls (N = 49440)**	**P-value**
Age - 0 ~ 30	3870 (39.14)	19329 (39.10)	0.9935
- 30-39	3695 (37.37)	18505 (37.43)	
- ≧40	2323 (23.49)	11606 (23.47)	
Gender -Female	1089 (11.01)	5445 (11.01)	1.0000
-Male	8977 (88.99)	43995 (88.99)	
Baseline comorbidity			
DM -Yes	194 (1.96)	792 (1.60)	0.0106
-No	9694 (98.04)	48648 (98.40)	
Drug abuse -Yes	316 (3.20)	129 (0.26)	<0.0001
-No	9572 (96.80)	49311 (99.74)	
Renal -Yes	34 (0.34)	85 (0.17)	0.0005
-No	9854 (99.66)	49355 (99.83)	
Liver Cirrhosis -Yes	83 (0.84)	124 (0.25)	<0.0001
-No	9805 (99.16)	49316 (99.75)	
Autoimmune -Yes	54 (0.55)	82 (0.17)	<0.0001
-No	9834 (99.45)	49358 (99.83)	

In Table 2, out of the total study sample of 9888 HIV-infected patients, 222 individuals (57.01/per 10000 person-years) developed DNI during the mean 3.94-year follow-up period. Out of the control sample of 49440 subjects without HIV, 735 individuals (35.54/per 10000 person-years) developed DNI. With the exception of the follow-up beyond 3 years (IRR = 1.26, *P* = 0.1255, *n* = 53 cases) after the HIV infection was diagnosed, the risk of developing DNI was significantly elevated in the HIV-infected groups in all ages, genders, and follow-up time frames.

**Table 2:** Risk of infection and soft tissue abscess for HIV patients and controls

**Characteristics**	**HIV**				**Controls**				**IRR (95 % CI)**	**P**
	**N**	**infection**	**PY#**	Rate*	**N**	**infection**	**PY#**	Rate*		
All	9888	222	38937.96	57.01	49440	735	206793.00	35.54	1.60 (1.38-1.86)	<0.0001
Age										
0 ~ 30	3870	92	16050.86	57.32	19329	277	82632.95	33.52	1.71 (1.35-2.16)	<0.0001
30 ~ 40	3695	71	14457.23	49.11	18505	251	76485.96	32.82	1.50 (1.15-1.95)	0.0027
γ 40	2323	59	8429.87	69.99	11606	207	47674.09	43.42	1.61 (1.21-2.15)	0.0012
Gender										
Male	8799	194	34755.23	55.82	43995	650	184421.29	35.25	1.58 (1.35-1.86)	<0.0001
Female	1089	28	4182.73	66.94	5445	85	22371.71	37.99	1.76 (1.15-2.71)	0.0093
Follow-up years										
<1	9888	71	9519.69	74.58	49440	179	49317.37	36.30	2.05 (1.56-2.70)	<0.0001
1-2	9422	54	9351.26	57.75	49198	167	49088.12	34.02	1.70 (1.25-2.31)	0.0007
2-3	9296	44	8309.14	52.95	48972	159	43951.53	36.18	1.46 (1.05-2.04)	0.0253
≧3	7231	53	11757.88	45.08	38481	230	64435.98	35.69	1.26 (0.94-1.70)	0.1255

#PY, person-years.

*Rate: per 10000 person-years.

The incidence rate and relative risk of DNI in the HIV-infected patients were 74.58 (per 10000 person-years) and 2.05 (*P* < 0.0001), and both were highest in the first follow-up year and then decreased on a yearly basis for the remainder of the follow-up. Beyond the 3-year follow-up, the incidence rate and the IRR were even lower at 45.08 (per 10000 person-years) and 1.26, which was not significantly elevated (*P* = 0.1255) as compared to the control group.

The details of the crude and adjusted HRs for DNI, based on the Cox proportional hazard regression analysis, are also carried out by cohort. After adjusting for the patient’s age, gender, drug abuse, chronic renal failure, liver cirrhosis, autoimmune disease, and diabetes mellitus, the HR for developing DNI during the mean 3.93-year follow-up period was 1.59 (95% CI, 1.358–1.862; *P <* 0.00001) for patients with HIV as compared to patients in the comparison cohort.

**Figure 1:**
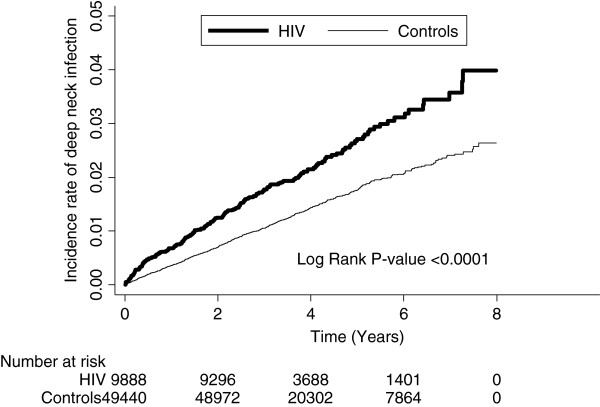
Incidence rate of DNI for HIV patients and controls.

**Figure 2:**
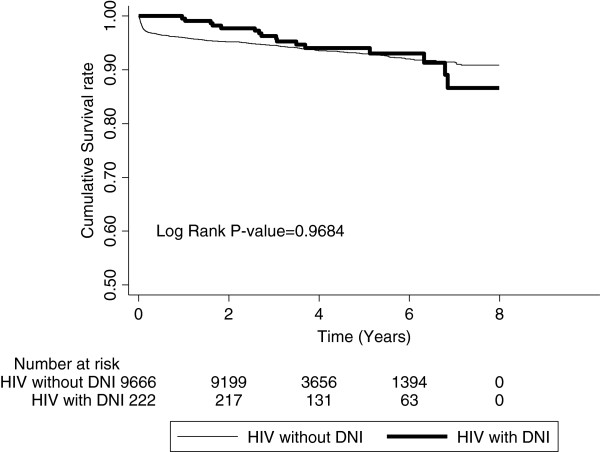
Survival rate for HIV-infected patients in the DNI and non-DNI groups.

The Kaplan-Meier analysis indicated that patients with HIV had a significantly higher incidence rate of DNI than the patients in the comparison cohort (*P* < 0.0001). The results of this analysis are presented in Figure 1. The Kaplan-Meier analysis was also used to estimate the survival rate for HIV-infected patients in the DNI and non-DNI groups. There was no significant decrease in the survival rate of the patients with HIV who had suffered from DNI attacks (*P* = 0.9684) as can be seen in Figure 2. There was a significant difference on the losses of follow-up in the first 2 years between the study cohorts and control cohorts in Table 3.

**Table 3:** Losses of follow-up for HIV patients and controls

**Interval**	**HIV (N = 9888)**		**Controls (N = 49440)**		P-value**
	N*	**Number of losses follow up**	N*	**Number of losses follow up**	
0-1	9888	395 (3.99)	49440	63 (0.13)	<0.0001
1-2	9422	72 (0.76)	49198	59 (0.12)	<0.0001
2-3	9296	2021 (21.74)	48972	10332 (21.10)	0.1645
3-4	7231	3522 (48.71)	38481	18064 (46.94)	0.0058

*N: Number of beginning.

** p-value obtained by chi-square.

## Discussion

Our results indicated that there may be more than just sporadic cases of DNI among HIV-infected patients, since the risk of DNI after an HIV attack was significantly elevated even with free access to HAART. In our study, the overall incidence of DNI in HIV-infected patients in Taiwan was 57.01 (per 10000 person-years), the first time this has been estimated in the world. The incidence risk ratio as compared to the control group was 1.60. After adjusting for the confounders, the HR was 1.59 (*P* < 0.0001). After adjusting for the other risks, we found that the risk of having a deep neck infection increased by 59% post-HIV infection. The log rank test indicated that patients with HIV had a significantly higher incidence rate of DNI than the patients in the comparison cohort (*P* < 0.0001).

In this study, the mean observation duration was 3.94 years (range: 2–8 years). This duration of time was sufficient to observe the trend and change in the risk of DNI among the HIV-infected patients under HAART. If we compare the incidence rate by follow-up years in the study group with the control group, the incidence rate among patients without HIV infection was relatively stable at about 35.55 ± 1.53 (per 10000 person-years).

After having HIV, the incidence rate for DNI was 74.58 (per 10000 person-years) and the incidence risk ratio was 2.05 (*P* < 0.0001) in the first follow-up year. Both the incidence rate and incidence risk ratio were highest in the follow-up years. In theory, the incidence of DNI should increase gradually, following the viral replication and CD4+ cell decline of the HIV-infected patients. It is interesting that the incidence rate and relative risk of DNI gradually decreased in the HIV-infected patients throughout the follow-up period. As time passed, the incidence risk ratio became lower and lower. Beyond the 3-year follow-up, the incidence of DNI and risk ratio were still higher than the control group, but they were lower than the previous 3 years, even down to 45.08 (per 10000 person-years) and 1.26 (*P* = 0.1255), which was not significantly elevated as compared to the control group.

HAART could play a role in this unanticipated result. Although we can hypothesize that this result is largely due to the well-known immune restoration consequences of HAART [15], we also need to consider the other cofactors implicated in this reduced incidence of DNI. HIV-infected patients are apt to develop aggressive periodontal disease and the associated ulcerative gingivitis, which can result in marked gingival recession and other oral lesions [16]. The increased risk of DNI could be the result of HIV damaging the immune system and decreasing resistance to oral infections, which was then further compounded by poor oral hygiene.

Even immune suppression for HIV-related cancer and related treatments may be a predisposing factor for DNI among HIV-infected patients. HAART has had a major impact on the reduction of plasma HIV RNA and the increase in peripheral CD4+ cells. It can also reduce the incidence of several opportunistic infections, including odontogenic infections and malignancies, especially AIDS-defining cancers [17,18]. HAART may have the capacity of effectively reducing the incidence rate of DNI.

Because of the side effects and the need for taking HAART continuously at the outset, not all HIV-infected patients accept HAART immediately after their HIV diagnosis. The timing of the initiation of HAART and the regimens are based on the guidelines recommended in the United States [19]. Following the gradual decrease in CD4 and increase in the virus, HIV-infected patients generally conform to the criteria and accept HAART. With more and more HIV-infected patients accepting HAART, the incidence of DNI and its relative risk should gradually decline to a steady level. This trend and the unexpected result suggested that HAART may decrease the risk of DNI among HIV-Infected patients.

Another possible consideration for the decrease in the incidence of DNI in the follow-up years may be the fact that the HIV patients whose immune systems were the most compromised did not survive, thereby decreasing the number of patients with the more severe immunity issues. We tried to evaluate the difference in the survival rates between the HIV patients with a history of DNI and the HIV patients without a history of DNI in order to rule out this hypothesis. In our study, there was no significant decrease in the survival rate of the patients with HIV who had suffered from DNI. On the other hand, there was a significant difference on the losses of follow-up between the study cohorts and control cohorts, so the time-decline in DNI rates on the HIV cohort may be explained by differences on the losses of follow-up in this study. Because of such a limitation, additional research is needed to examine the role of HAART in reducing the risk of DNI among HIV-infected patients.

The use of administrative data to assess the contribution of HIV infection to DNI is subject to several biases such as information bias and selection bias. For example, it is well known that patients with drug abuse have increased rates of periodontitis. It may cause the selection bias which might affect the result of the study. In order to avoid such a bias, we took the diseases which could be predisposing factors of DNI in previous studies including drug abuse as comorbidities for both the study and comparison cohorts. After adjusting for the patient’s comorbidities, the effect of the bias which had affected the HR could be minimized. Likewise co-infection by HPV in patients having sex with men may increase the rate of cancer of oral cavity. If it is the case, the risk of oral cancer might increase significantly in the patients with HIV in comparison to the controls. However, the incidence rate ratio between HIV-infected patients and the controls for oral cancer was 1.23 (95%CI = 0.63-2.41) in the present study. Hence, the effect of confounding variable of HPV infection in present study remains to be proved.

There were several limitations in the present study. First, the diagnosis of DNI was based on the diagnostic code registered by the physicians responsible for the treatment of the patients and was not corroborated by the investigators. Second, the risk of DNI after an HIV attack was severely underestimated, because all of the HIV-infected patients in this study were offered free access to HAART. The real risk of DNI among HIV-infected patients would need to be estimated with untreated patients, but political and ethical reasons prevent such a study in Taiwan. Nonetheless, we still successfully proved the elevated risk of DNI among HIV-infected patients even when they had free access to HAART. The influence of HAART on HIV-infected patients should be compared using untreated patients, but, of course, political and ethical reasons again prevent this study. Fortunately, the long-term trend could be clearly observed in this study. Additional investigations on this issue are needed. Third, although the Taiwanese government will provide all HIV-infected citizens with free access to HAART, the HIV-infected patients make their own decisions as to when to start HAART. The degree of compliance of the patients using HAART was also not covered in this study.

## Conclusions

In conclusion, we believe this is the first attempt at investigating epidemiological data on DNI among HIV-infected patients in a nationwide population-based study. The results of the present study indicate the risk of DNI is significantly elevated among HIV-infected patients, even with free access to HAART. Additional research is needed to examine the role of HAART in reducing the risk.

## Competing interests

The authors’ declare that they have no competing interests.

## Authors’ contributions

CF Liu designed the study and drafted the manuscript. CF Lien participated in the design of the study and helped to draft the manuscript. SFW contributed to the interpretation of the data, and performed the statistical analysis. YSL, CSL and JJW contributed to the acquisition of the data and revising it critically. All authors read and approved the final manuscript

## Pre-publication history

The pre-publication history for this paper can be accessed here:

http://www.biomedcentral.com/1471-2334/13/183/prepub
